# Cost-Effectiveness of Robotic vs. Laparoscopic Surgery for Different Surgical Procedures: Protocol for a Prospective, Multicentric Study (ROBOCOSTES)

**DOI:** 10.3389/fsurg.2022.866041

**Published:** 2022-05-06

**Authors:** Benedetto Ielpo, Mauro Podda, Fernando Burdio, Patricia Sanchez-Velazquez, Maria-Alejandra Guerrero, Javier Nuñez, Miguel Toledano, Salvador Morales-Conde, Julio Mayol, Manuel Lopez-Cano, Eloy Espín-Basany, Gianluca Pellino, The ROBOCOSTES Study Collaborators, Dulce Momblan

**Affiliations:** ^1^Hepato-Biliary and Pancreatic Surgery Unit, Department of Surgery, Hospital del Mar, Barcelona, Spain; ^2^Department of Surgical Science, Emergency Surgery Unit, University of Cagliari, Cagliari, Italy; ^3^IVEC (Instituto de Validación de la Eficiencia Clínica), Fundación de Investigación HM Hospitales, Madrid, Spain; ^4^General Surgery Department, University Hospital Rio Hortega, Valladolid, Spain; ^5^Unit of Innovation in Minimally Invasive Surgery, Department of Surgery, University Hospital Virgen del Rocio, University of Seville, Seville, Spain; ^6^Department of Surgery, Hospital Clinico San Carlos, Universidad Complutense de Madrid, Madrid, Spain; ^7^Abdominal Wall Surgery Unit, Vall d'Hebron University Hospital, Universitat Autónoma de Barcelona, UAB, Barcelona, Spain; ^8^Colorectal Surgery, Vall d'Hebron University Hospital, Universitat Autónoma de Barcelona, UAB, Barcelona, Spain; ^9^Department of Advanced Medical and Surgical Sciences, Università degli Studi della Campania “Luigi Vanvitelli”, Naples, Italy

**Keywords:** ROBOCOSTES study protocol robotic surgery, laparoscopic surgery, cost-effectiveness, QALY, multicenter studies

## Abstract

**Background:**

The studies which address the impact of costs of robotic vs. laparoscopic approach on quality of life (cost-effectiveness studies) are scares in general surgery.

**Methods:**

The Spanish national study on cost-effectiveness differences among robotic and laparoscopic surgery (ROBOCOSTES) is designed as a prospective, multicentre, national, observational study. The aim is to determine in which procedures robotic surgery is more cost-effective than laparoscopic surgery. Several surgical operations and patient populations will be evaluated (distal pancreatectomy, gastrectomy, sleeve gastrectomy, inguinal hernioplasty, rectal resection for cancer, Heller cardiomiotomy and Nissen procedure).

**Discussion:**

The results of this study will demonstrate which treatment (laparoscopic or robotic) and in which population is more cost-effective. This study will also assess the impact of previous surgical experience on main outcomes.

## Background

The advantages of minimally invasive approach in general surgery procedures have been reported in many studies (1). Robotic surgery provides three-dimensional vision, seven motion degrees, stable vision, disabling hand's tremor, and a well-established feasibility, safety, and effectiveness profile (1–3).

While randomized controlled trials and observational studies have demonstrated the safety and effectiveness of robotic surgery, they rarely assess its economic impact on the healthcare system compared with the laparoscopic technique (4–7). This needs to be addressed but planning such analyses before starting the study is essential and most of the times was not properly measured.

## Current Evidence

Several studies have shown that robotic-assisted surgery is associated with higher costs than laparoscopy (6–8). Contributing factors include purchasing the robot by the healthcare institution, maintenance costs, disposable materials, and longer operative times. These apply to most general surgery procedures; but some organ- and procedure-specific considerations are needed.

Regarding distal pancreatectomy, two recent meta-analyses aiming to compare the costs of robotic and laparoscopic distal pancreatectomy, demonstrated that the latter seems to contain costs, despite some potential technical advantages of the robotic technique (e.g., decreased conversion rate) (8, 9). However, the surplus cost of robotic distal pancreatectomy resection might be reduced given the decrease in hospitalization costs.

Regarding rectal surgery, six studies in the literature reported on the costs of robotic vs. laparoscopic rectal resection (7, 10–14). According to Morelli et al., hospital costs were slightly higher in the laparoscopic group, whereas higher operative costs were reported in the robotic rectal resection group (11). Baek et al. reported overall costs to be higher in the robotic group, and data on hospitalization costs were found to be generally lacking (13).

Some published result must be interpreted with caution as the analysis is based on heterogeneous data with outcomes conditioned by the lack of an economical standardization of reported costs. A study from 2014 reported that robotic-assisted colorectal resections had higher total ($5,272 increase, *p* < 0.001) and direct costs ($4,432 increase, *p* < 0.001), longer operating time (39 min, *p* < 0.001), and were more frequently associated to postoperative bleeding (OR 1.6, *p* = 0.014) than laparoscopic resections. The data source for this study was a United States national inpatient database evaluating a total of 17,265 laparoscopic and 744 robotic colorectal resections between 2009 and 2011 (10). However, there were some limitations in this study that may have influenced the results. First, the authors used an administrative database which had the possibility of coding errors. Second, many centers were involved, which also suggests a high heterogeneity of the techniques in both the laparoscopic and robotic groups, including the type of devices, the implementation of hybrid vs. totally robotic techniques, the phase of the learning curve, and patient volume at the center. Furthermore, some data were missing, such as the conversion rate.

The main reasons why the costs of robotic approach are poorly reported in the literature are mainly due to the challenge in their calculation. If, from one hand, costs associated with the hardware are easy to obtain, the impact of the surgical procedure on overall costs is difficult to be quantified.

Indeed, overall costs should include not only operative costs but also costs related to the rehabilitation facility, days out of work after surgery and its impact on the quality of life (QoL), which make an exact estimate challenging. Studies which address the impact of costs of a new technique on QoL are named *cost-effectiveness studies*.

## The Rationale for the Study

The current debate about introducing a new technology mainly concerns its cost-effectiveness (15). Only a cost-effectiveness analysis can shed light on uncertainties concerning the economic impact of robotic vs. laparoscopic surgery. However, reliable, prospective, adequately designed cost-effectiveness studies are still lacking (16–21).

QoL after a surgical procedure can be measured through different scores (22). In order to conduct cost-effectiveness studies, QoL needs to be objectively and consistently measured, using validated tools. The most used scores to grade QoL are the generic SF-36 and the EuroQol EQ-5D-5L tests, that can be transformed into QALYs, giving a more objective view of the patient's improvement after the planned treatment (health perspective) (23).

Apart from prospective series, some studies only used a decision-analytic model derived from available literature for costs, QoL and outcomes (24). However, these results must be interpreted with great caution since the insufficient data available in the literature imply a high risk of bias.

According to the pyramid of evidence, randomized clinical trials represent the highest level of study types. However, in a surgical setting of robotic vs. laparoscopic approach, these trials are difficult to be performed (e.g., expensive, ethical issue, blinding) and generalized (strict inclusion criteria) (25). Hence, prospective, adequately designed and conducted non-randomized clinical trials can provide very clinically relevant information.

A well-designed cost-effectiveness study would answer whether its superiority justifies the higher costs of robotics in terms of gained QoL after surgery. This protocol describes the study of nationwide assessment of the cost-effectiveness of the laparoscopic vs. robotic approach, which will define which approach is more convenient for each condition or patient.

## Aims of the Study

### Primary Aim of the Study

To determine if robotic surgery is more cost-effective than laparoscopic surgery in several surgical conditions and patient populations. The present prospective study will evaluate the incremental cost per quality-adjusted life year (QALY) gained from healthcare perspectives.

### Secondary Aims of the Study

The secondary aim is to explore the difference between groups concerning assessment of efficacy (hospital stay, pain, proportion and time to uptake of chemotherapy), and measures of safety (adverse health events).

## Study Design

The Spanish national study on cost-effectiveness differences among robotic and laparoscopic surgery (ROBOCOSTES) is designed as a prospective, multicentre, national, observational study conducted in Spain between the 15th of January and the 15th of December 2022.

### Study Population

Patients are considered eligible to enter the study if they meet the following inclusion criteria:

1. Adults aged ≥18 years of age.

2. Consecutive patients undergoing: distal pancreatectomy, gastrectomy (total and subtotal), sleeve gastrectomy, inguinal hernioplasty with mesh, rectal resection for cancer, Heller cardiomiotomy and Nissen procedure.

3. Type of disease approachable by both laparoscopic and robotic minimal access surgery.

### Exclusion Criteria

The following exclusion criteria will apply:

1. Patients with peritoneal metastases.
2. Patients with ASA physical status IV-V.
3. Emergency surgery: that is required to deal with an acute threat to life.
4. Inability to give written informed consent.
5. Surgery for recurrent disease of the included procedure.
6. Palliative surgery: performed only for symptom relief even despite negligible impact on the patient's survival.

## Quality Control

### Center Registration

Centers will be allowed to upload data after the registration procedure. Local ethics committee approval is needed, and each study local lead will be responsible for local study approval before the study start. Each center will register a principal investigator and a co-investigator for each type of procedure eligible for the study.

### Surgeon Registration

Invitation emails will be sent to every associated member by the AEC. Data about hospital characteristics (academic, teaching, referral, or country hospital, number of beds) and surgical volumes both for robotic and laparoscopic surgery will be registered using a Google Form survey (Google, Mountain View, California, USA): https://forms.gle/eHCELgr5nZCRwUgP9. Each surgeon introducing data and participating in the study will be asked about his previous experience in both laparoscopic and robotics concerning the selected procedure.

## Data Collection

### Clinical Data

Clinicians will be requested to register medical data related to surgery (operation equipment, skin-to-skin operating time, blood loss, surgical equipment, number of surgeons), hospital stay (intensive care unit, high dependency unit, ward), complications and associated management (interventions, additional imaging, additional hospitalization). This data will be entered into an electronic Case Report Form (eCRF) online database (CASTOR).

Complications will be classified according to the Clavien-Dindo classification of surgical complications (26) and registered as adverse or serious adverse events. Participants' overall morbidity will be assessed using the Comprehensive Complication Index (CCI) (27). The 95% confidence intervals (CI) for the difference in the means between the two groups will be reported as a primary result. Since the CCI is approximately normally distributed, the 95% CI can be computed using the normal distribution. Further descriptive measures will be reported in terms of mean and standard deviation.

Missing data will thus be managed depending on the cause of missingness. If missingness may directly or indirectly be related to the treatment, it may cause a bias even in the intention-to-treat analysis. In such a situation, the missing data will be reported in the final report, and missing data in the intention to treat analysis will be analyzed according to the worst-case scenario method (failure).

## Data Completeness

### Questionnaires

All participants will be asked to provide an additional informed consent to receive the validated QoL questionnaires EuroQol EQ-5D-5LTM (official Spanish version) and will be asked to score five aspects of health status using the EQ-5D™ questionnaire: mobility, self-care, usual activities, pain/complaints, and mood (anxiety/depression). This questionnaire is considered the most representative QoL tool for cost-effectiveness studies (28). Patients will receive questionnaires at inclusion, one, and 3 months after surgery, by mail, digitally, or in person, according to their preference.

### Cost Data

Data on costs will be collected from the Spanish Hospital Costs Network (*Red Española de Costes Hospitalarios, RECH* https://www.rechosp.org/rech/faces/en/jsf/index.jsp). RECH is an official Spanish project initiated to obtain an extensive database to provide reliable information on the per-patient cost of hospital care. The RECH database is a healthcare database that brings together the results of different management efforts in many Spanish Healthcare System institutions. Overall direct hospital costs will be collected, except for the costs associated with the acquisition or maintenance of the robotic device. Pre-admission charges for screening will not be considered, as these are equal for both surgical techniques. All readmission-related costs will be added to the total hospital expenses. A discount rate of 3% per year will be used to estimate the costs and QALYs, as recommended by health economic guidelines (28). All costs will be presented in Euros (exchange rate 2022).

## Auditing

Independent qualified monitors will monitor the study. Data will be entered into an electronic eCRF, and after the monitoring, visits will be marked as “complete data.” Monitoring visits will be scheduled according to the number of visits ready for verification. The date of the visit will be agreed upon with centers in advance. Before initiation of the trial, interactive training will be conducted, and an electronic test database will be created for familiarization with the system and test data entry. Data's completeness, validity, and plausibility will be checked at the time of data entry (edit checks) and by using validating programs that generate queries. The completed eCRF must be reviewed and signed by the investigator named in the trial protocol or a designated sub-investigator. The investigator or the designated representative will be obliged to complete the eCRF as soon as possible after information is collected and to clarify or explain the queries.

## Sample Size Calculation and Statistical Methods

### Sample Size

There are concerns about calculating power and sample size in cost-effectiveness studies (28). Although there is some uncertainty, the Incremental Net Benefit (INB) is a measure that is easier to interpret and work with, since a positive INB means that the new proposed treatment is cost-effective (29). Medicare calculations of power and sample size are calculated against some value of Maximum Willingness to Pay (WTP) for a unit of treatment effect. The WTP is defined from a societal perspective, and, although in Spain there is no specific WTP threshold in healthcare, according to the National Institute for Health Care Excellence (NICE) (30) and Spanish Recommendations on Economic Evaluation of Health Technologies (31) we use a WTP of 20,000 € and 30,000 € per QALY as a threshold, to recognize which treatment is most cost-effective, as follow:


$$\begin{array}{l} \text{INB}=\left[\left(\text{EffectNew-EffectComparatore}\right)*\text{WTP}\right]\\ -\left(\text{CostNew-CostComparator}\right) \end{array}$$

This sample size calculation was realized with consultation of two statisticians.

Considering previous retrospective reports on cost-effectiveness studies (16–21) in which a minimum of 3,000 € for QALY gained was considered cost-effective, assuming a 5% lost-to-follow-up-rate and the two-sided significance level (α) set at 5% and power (1-β) at 80%, a minimum total of 60 patients need to be allocated in each procedure group comparison (30 laparoscopic vs. 30 robotic).

### Statistical Method

Data will be analyzed using the SPSS statistical program (SPSS Inc. Chicago, IL, USA). In order to compare the means of the quantitative variables when these followed a normal distribution, a variance analysis and Student's *t-*test will be used. For the rest of the variables, both the Mann-Whitney and Kruskal-Wallis tests will be performed. For categorical variables, a Chi-square test will be implemented. Results with a *P*-value lower than 0.05 will be considered statistically significant.

### Cost-Effectiveness Analysis

The economic evaluation will be performed as cost effectiveness and cost–utility analyses from a healthcare perspective, with a time horizon of 3 months.

The cost–utility endpoint will be the cost per QALY. The results will be reported in accordance with CHEERS guidelines (23). Data will be collected in the early post-operative period of time (3 months from the surgery) in order to capture as much as possible only the QoL mainly related to the surgical approach instead of the issues related to all treatment process (adjuvant treatment, stoma issues, etc.) which patients are dealing with in a later period of time.

An independent company (Institute for Validation of Clinical Efficacy - IVEC) will perform the financial analysis, eliminating the risk of an observer bias.

A model-based cost-utility analysis estimating mean costs and QALYs per patient will be performed. A stochastic cost-utility analysis will be undertaken. The incremental cost-effectiveness ratio (ICER) will be estimated using the overall costs of the robotic and laparoscopic procedures and QALYs derived from patient interviews to find the incremental cost per QALYs gained.

The INB will be calculated to estimate decision-makers maximum willingness to pay (WTP) for a QALY gained. The INB will be calculated as the mean QALYs per patient multiplied by WTP threshold minus the mean cost per patient for the treatment. The decision rule is to adopt the treatment if the INB > 0, and the alternative with the highest INB represents best value for money.

### Sensitivity Analysis

A sensitivity analysis will be implemented to propagate the uncertainty of the estimations to the model's results. We will use a multivariate and stochastic sensitivity analysis performed by 5,000 Monte Carlo simulations. The cost-effectiveness plane will represent all pairs of solutions of the model.

The results of the one-way sensitivity analysis will be reported in the tornado diagram, which depicts graphically how variations in each input affect the outcome. In addition, the 95% CI intervals around the base case values will be derived using the 2.5 and 97.5 percentiles calculated from the sensitivity analysis.

The tornado diagram will be stacked in order of decreasing width, indicating that variations in inputs near the top (Total Costs of Robotics procedures) have the most significant effect on the outcome, while variations in inputs near the bottom (QALYs discount rate) have relatively small effects on the outcome.

### Acceptability Curve

The analysis will also include a cost-effectiveness acceptability curve that plots the probability that the robotic technique is cost-effective relative to laparoscopy over a reasonable range of levels of WTP.

Although there is no specific willingness to pay threshold in healthcare in Spain, as described above, according to the National Institute for Health Care Excellence (NICE) (30) and the Spanish Recommendations on Economic Evaluation of Health Technologies (31) a WTP of 20,000 € and 30,000 € per QALY will be used as a threshold to recognize which treatment is most cost-effective.

## Methods for Minimizing Bias

The number of patients screened, the number included, and those analyzed will be reported, and differences will be explained. In addition, the patient flow and the Consolidated Standards of Reporting Trials (CONSORT) flowchart will be reported in the final analysis (32).

In addition, analysis will be stratified according to each surgeon's previous experience before starting the study, type of hospital (public, university) and total number of beds.

## Interim Analysis

An interim analysis of the primary outcome will be performed after inclusion of 50% of the sample.

## Ethics

### Research Ethics Approval

The study has been registered in ClinicalTrials.gov (ID NCT04861974). The Local Ethical Committee of the Coordinating Center (Parc Salut Mar Hospital, Barcelona) has approved this study with the code: *2020/9514/I* and institutional review boards of the participating centers will be requested to provide their approval. Furthermore, the study is approved by the Spanish Surgical Association committee which funded this study with its budget.

### Consent and Assent

Informed consent will be obtained by the treating surgeons in each participating center. In addition, patients will be allowed to provide separate permission for collecting blood and/or tissue samples for translational research and for receiving the QoL questionnaires.

### Confidentiality

Individual patient information obtained from this study will be considered confidential, and their handling will conform with the Spanish policy on data protection. In addition, patients' confidentiality will be ensured by using study numbers.

## Ethics Statement

The studies involving human participants were reviewed and approved by IMIM. The patients/participants provided their written informed consent to participate in this study.

## Author Contributions

BI contributed to conception and design of the study. GP, MP, FB, and PS-V organized the database. BI, JN, and MT wrote the first draft of the manuscript. SM-C, JM, ML-C, and EE-B wrote sections of the manuscript. All authors contributed to manuscript revision, read, and approved the submitted version.

## Funding

Project PI20/00008, funded by Instituto de Salud Carlos III (ISCIII) and co-funded by the European Union.

## ROBOCOSTES Study Collaborators

Dulce Momblan, Sandra Castro Boix, Esteban Cugat, pardo, Marcel Pujadas, Rosa Jorba, Juan Bellido Luque, Carmen Cagigas, Jacobo Trebol, Juan Carlos Martín del Olmo, Helena Álvarez García, José Herreros Rodríguez, Silva Fernández, Luis Sanchez Guillen, José Francisco Noguera Aguilar, Raquel Sánchez Santos, Juan José Segura Sampedro, M^*a*^ Asuncion Acosta Mérdia, Iván Jesús Arteaga González, Irene Ortega Vázquez, Mario Álvarez Gallego, Emilio Vicente, Sagrario Martinez Cortijo, Sergio Pedro Olivares Pizarro, David Fernandez Luengas, David Alías Jiménez, Carolina González-Abós, Amaia Gantxegi, Clara Codony Bassols, Isaias Alarcon del Agua, Carlos Rafael Díaz Maag, Aroa Abascal Amo, Carlos Jezieniecki Fernández, David Pacheco Sánchez, Julián García Orozco, Beatriz Guil Ortiz, Manuel Alberto Lasaia, Raquel Ríos, Jorge Zárate Gómez, Gustavo Díaz García, Víctor Rodrígues, Guillermo Ais Conde, Esther Ferrero Celemín, Santiago Linacero, Miguel Josa, Marta Pascual, Miquel Kraft Carré, Pere Planellas Giné, Javier Sánchez González, Alberto Rojo López, Rita Medina Quintana, Ana Pilar Morante, Fabio Ausania, Elisabeth Pando, Santiago López-Ben, Ángel Cuadrado García, José Antonio Pereira, Alex Bravo, Juan Beltrán de Heredia Rentería, Daniel Sánchez López, Anna Casajoana, Ramón Vilallonga Puy, Luis García Sancho, María Hernández O‘Reilly, and José María Gil López.

## Conflict of Interest

The authors declare that the research was conducted in the absence of any commercial or financial relationships that could be construed as a potential conflict of interest.

## Publisher's Note

All claims expressed in this article are solely those of the authors and do not necessarily represent those of their affiliated organizations, or those of the publisher, the editors and the reviewers. Any product that may be evaluated in this article, or claim that may be made by its manufacturer, is not guaranteed or endorsed by the publisher.
